# Stercoral Perforation in the Setting of Chronic Antipsychotic Use: A Case Report

**DOI:** 10.7759/cureus.33631

**Published:** 2023-01-11

**Authors:** Masha Osman, Michele Fiorentino, Michael Gwiazdowski, James E Gervasoni

**Affiliations:** 1 General Surgery, St. George's University School of Medicine, New York, USA; 2 Department of Surgery, Vanderbilt University Medical Center, Nashville, USA; 3 Department of Internal Medicine, Nuvance Health, New York, USA; 4 Department of Surgery, Saint Peter’s University Hospital, New Brunswick, USA

**Keywords:** chronic constipation, chronic opioids, olanzapine, antipsychotics, stercoral perforation

## Abstract

Stercoral perforation is a rare form of colonic perforation with limited reports in the literature, accounting for less than 140 documented cases. This complication occurs due to increased intraluminal pressure created by fecal impaction, ultimately causing colonic ulceration and necrosis. It is most often seen in elderly or debilitated patients with chronic constipation. The long-term use of drugs or medications with side effects of chronic constipation such as opioids, antispasmodics, tricyclic antidepressants, and calcium channel blockers have been implicated in these cases. Here we present a case of stercoral perforation in a patient with short-term opioid use following an orthopedic procedure, but more likely complicated by long-term use of antipsychotics and antidepressants.

## Introduction

Stercoral perforation is associated with chronic constipation, the development of a stercoral ulcer, and ultimately perforation. Although rare, mortality rates in patients with stercoral perforation are as high as 34% [1]. These ulcerations and perforations are most common at the anterior rectum, the antimesenteric border of the rectosigmoid junction, and the sigmoid colon because of the physiologic characteristics of these segments. They normally comprise a lower water content of stool, poor blood supply, and high pressure due to a narrowed intraluminal diameter [2], all factors that predispose them to a much higher risk of perforation when associated with chronic constipation. Opioid use is most commonly implicated in the side effect profile of constipation, but other medications such as antispasmodics, antidepressants, antipsychotics, and calcium channel blockers are known to cause constipation as well. We present a case report where stercoral perforation occurred in the setting of short-term opioid use and long-term antidepressant and antipsychotic use, an uncommon presentation of an already rare event. This preempts further attention to be given to patients given opioids for pain control when they are already on medications that can cause chronic constipation, in order to reduce their risk of developing a stercoral perforation.

## Case presentation

A 59-year-old woman with a past medical history significant for hypertension, major depressive disorder, gastroesophageal reflux disease (GERD), and asthma presented to the emergency department with one week of diffuse abdominal pain that had acutely worsened overnight. She reported that her last bowel movement was five days prior and her last flatus was two days prior. A month earlier, she had undergone a left total knee replacement for which she had been taking oxycodone for pain control. Her home medications included albuterol, amlodipine, bupropion, desvenlafaxine, escitalopram, lamotrigine, lisinopril-hydrochlorothiazide, montelukast, olanzapine, omeprazole, and vilazodone. She denied any smoking, alcohol, or illicit drug usage.

On physical exam, the patient had a temperature of 98.6F, a heart rate of 88 beats/min, and blood pressure of 88/55 mmHg. She was alert and oriented but appeared to be in mild distress. On abdominal exam, she had peritonitis with diffuse tenderness and guarding. However, there was no obliteration of liver dullness. Physical examination was unremarkable for all other systems.

**Figure 1:**
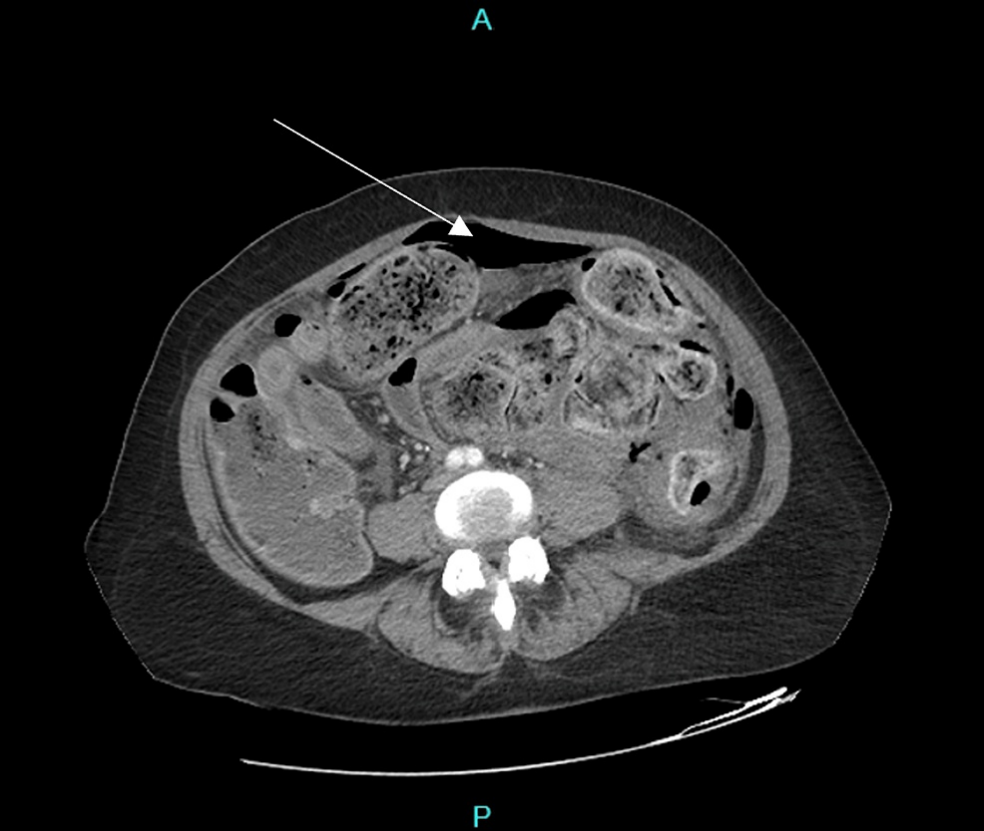
CT abdomen The image is showing peritoneal free air (arrow) and diffuse stool burden

**Figure 2:**
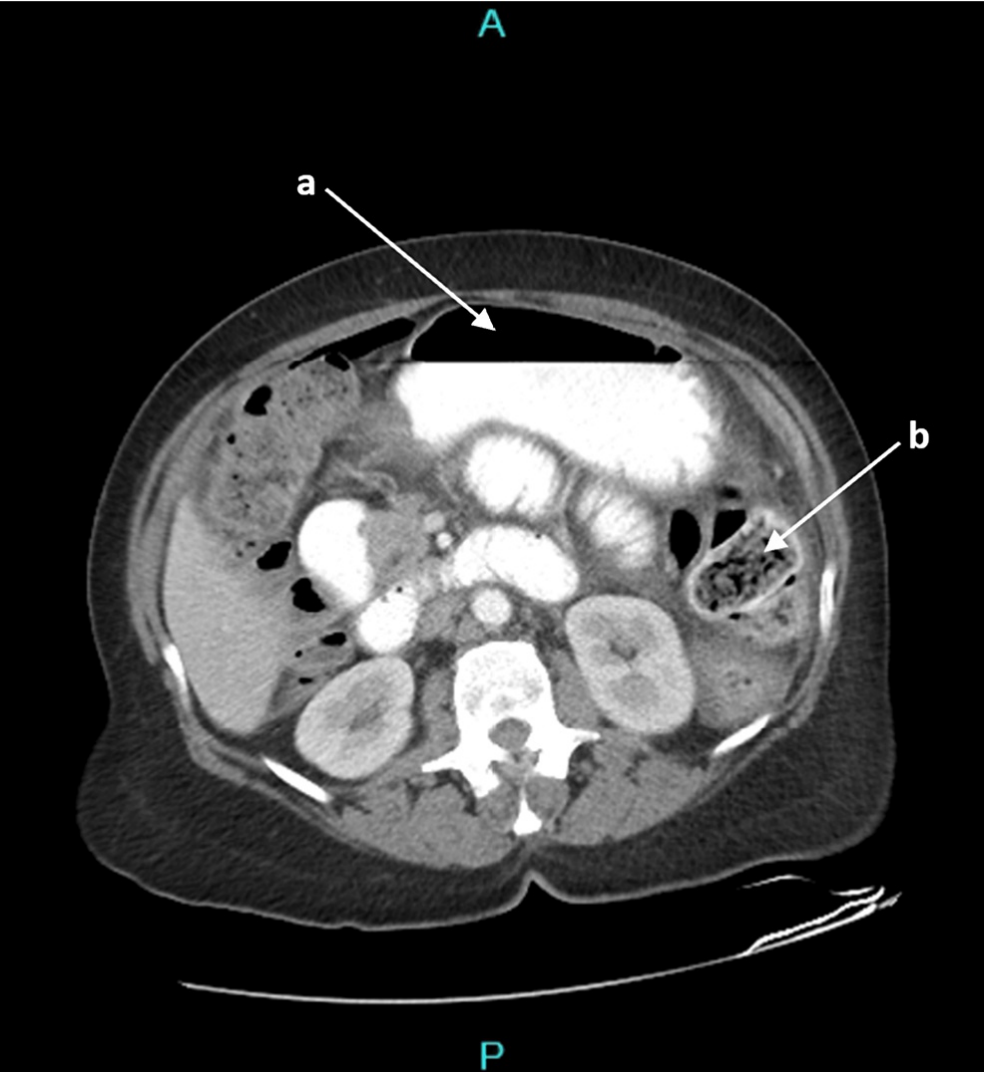
CT abdomen with oral contrast The image is demonstrating significantly distended loops of bowel with air-fluid level and stool burden. Arrow a demonstrates air-fluid level and bowel distention. Arrow b points to stool burden in one loop of bowel

Her laboratory values were significant for a white blood cell count of 4.9 × 10^9^/L, Hemoglobin of 11.5 g/dL, Sodium of 125 mmol/L, BUN of 9 mg/dL, Creatinine of 0.9 mg/dL, and lactate of 2 mmol/L. A Computed Tomography (CT) scan of the abdomen and pelvis showed peritoneal free air with diffuse stool burden and obstruction (Figures 1, 2) that was not adequately visualized on plain film radiography. Oral contrast was also given to better target the possible source of peritonitis, i.e. gastric/duodenal versus sigmoid/rectal and direct the best course of action during surgery.

Consequently, a nasogastric tube was immediately placed with 700 cc bilious output. The patient was emergently taken to the operating room for exploratory laparotomy. Upon entering the abdomen, murky fluid and free-formed stool were found. One bucket full of formed stool was removed from the abdomen. Examination of the bowel revealed a 5 cm wide perforation with complete dissociation of the sigmoid colon. Consequently, a sigmoidectomy was performed along with Hartmann’s closure of the rectal stump. A colostomy was created with a takedown of the splenic flexure in order to prepare the patient for adequate re-anastomosis in the near future. The patient recovered over the next several days and was discharged home without any further complications. Four months later, following a normal colonoscopy, the patient was able to undergo a reversal of the ostomy.

## Discussion

The prevalence of stercoral perforation of the colon (SPC) is rare, with only 137 cases reported in the literature since its discovery in 1894 [1]. Although rarely reported, the incidence is estimated to be much higher, encompassing around 1.2% of all emergency colorectal surgical procedures and 3.2% of all colonic perforations [3]. The criteria for stercoral perforation involve the following: 1) the colonic perforation must be round or ovoid, exceed 1 cm in diameter, and lie anti-mesenterial; 2) fecalomas are evident within the colon, protruding through the perforation site or lying within the abdominal cavity; and 3) pressure necrosis or ulcer formation and a chronic inflammatory reaction around the perforation site are present microscopically [3]. Additionally, it is important to note that these criteria are only applicable in the case of a colon free of a pre-existing disease or structural abnormalities. Our case fulfilled these criteria with a perforation measuring 5 cm, complete dissociation of the sigmoid colon, free stool in the abdominal cavity, and chronic inflammation present on the colon as per the pathology report.

These perforations are commonly related to chronic constipation and medications that induce constipation. There have been many documented cases of chronic opiate usage that result in SPC yet to our knowledge no such reports with short-term opiate use. There are reports of stercoral perforation that are associated with the long-term use of antipsychotics. Antipsychotics have been shown to have side effect profiles that include anticholinergic effects such as constipation as evidenced in The Clinical Antipsychotic Trials of Intervention Effectiveness (CATIE) study [4], where anticholinergic side effect panels were examined. In particular, olanzapine was identified to cause constipation in 24% of patients receiving treatment [5]. In addition, studies have found that olanzapine specifically has a 9-11% higher incidence of constipation as compared to other antipsychotics, as well as a dose-dependent increase. This increase in reported constipation appears to be related to the 5HT_3_ receptors in the gastrointestinal smooth muscle that regulate muscle contraction [6]. Similarly, in a case series by Haddad et al., stercoral perforation was reported in a patient who was on olanzapine for the treatment of schizophrenia [7].

In a patient who is taking multiple medications that converge on the side effect of constipation, adding another medication with a similar common side effect profile such as opioids compounds their risk of developing constipation. Our patient was on several medications including olanzapine, desvenlafaxine, escitalopram, and lamotrigine, all of which have been identified as having side-effect panels that include constipation [8-10]. As we have seen in this case and according to the criteria listed by Maurer et al. [3], a patient with seemingly normal bowel function and no underlying bowel disease can potentially go on to develop stercoral perforation. It would be beneficial in such cases to make an effort to assess the patient's prior history of bowel function and current list of medications, and to offer prophylaxis in the event that constipation does surface [11]. This is especially important in the setting of prescribing opioids for pain management due to their well-known effects on bowel motility and the ability to cause constipation and obstruction [11].

## Conclusions

This presentation of stercoral perforation fulfills the criteria needed to trigger such an event. Contrary to the literature however, this case does not relate to the more classical presentation of stercoral perforation through chronic constipation due to long term opiate usage. The patient’s chronic use of psychiatric medications in addition to short-term use of opioids likely combined to cause constipation and eventual stercoral perforation. When adding new medications such as short-term opioids, it is imperative to discuss side effect profiles with the patient, including constipation. In patients with high risk for constipation, a bowel regimen should be initiated prophylactically to prevent such complications from occurring.
